# Case report: Four cases of kidney disease in Giant African Land Snails (*Lissachatina fulica*)

**DOI:** 10.3389/fvets.2023.1152281

**Published:** 2023-05-15

**Authors:** Silvana Schmidt-Ukaj, Michaela Gumpenberger, Frank Mutschmann, Barbara Richter

**Affiliations:** ^1^Service for Birds and Reptiles, Small Animal Internal Medicine, Vetmeduni Vienna, Vienna, Austria; ^2^Clinical Unit of Diagnostic Imaging, Small Animal Internal Medicine, Vetmeduni Vienna, Vienna, Austria; ^3^Exomed-Institute, Berlin, Germany; ^4^Institute of Pathology, Vetmeduni Vienna, Vienna, Austria

**Keywords:** kidney disease, mollusks, invertebrates, diagnostic imaging, case report

## Abstract

Giant African Land Snails like *Lissachatina fulica* have become increasingly popular as pets in Europe, but little is known yet about land snail diseases and their therapy. These case reports show the history of four adult *Lissachatina fulica* with apathy and esophagus protrusion or prolapse in three cases and edema and mantle collapse in another case. Renomegaly and/or kidney deposits could be demonstrated in radiographic and/or CT examinations in all four cases. Necropsy and histology revealing nephropathy could be performed in three cases. With these first case reports on land snails with kidney disease, we hope to encourage diagnostic imaging and further veterinary research in land snails to improve our knowledge about their diseases.

## Introduction

Giant African Land Snails (GALS) have become increasingly popular as pets in Europe, although they are regarded as economically important snail pests in many countries of the world. Taxonomically, these snails belong to the family of *Achatinidae*, to the taxonomic clade of *Eupulmonata*, the class of *Gastropoda* and the phylum *Mollusca*^1^ and they are native to Africa.

These pulmonated snails have an unichambered shell protecting the visceral organs, a head with two pairs of tentacles including the eyes and the olfactory organs, as well as the mouth and a ventrally situated muscular foot (1, 2). Different species of GALS are held and bred in captivity. One of the most popular is *Lissachatina fulica* (*L. fulica*).

Despite being occasionally presented as veterinary patients, little is known and recorded about their diseases and therapy. Cooper and Knowler (3) gave an overview about basic biology, husbandry, handling, diseases, anesthesia, and euthanasia in land snails. Possible risk factors for several diseases included failures in husbandry and feeding as well as trauma, poison, parasites, bacterial, and fungal infections. Kidney diseases, prolapse, edema or mantle collapse were not mentioned. Clinical techniques for invertebrates including land snails were additionally described by Braun et al. (4). Diseases in gastropods were also investigated by Smolowitz et al. (1), however their focus laid on marine snails and parasitic kidney diseases were described. In *Achatinidae* only “shell disorders” were mentioned.

Interestingly these authors also provided an imaging part with a short literature review of imaging techniques in aquatic gastropods. Another comprehensive imaging study in mollusks is available by Ziegler et al. (5). These authors used different computed tomography (CT) and magnetic resonance imaging (MRI) techniques in aquatic mollusks like *Lepidochitona cinerea*, *Acanthochitona crinita* or *Sepia officinalis* to study morphology and anatomy. Only one study provides a description of the physiological anatomy of land snails of the family of *Achatinidae* as a reference for radiographic, sonographic, and CT examinations and determined which diagnostic imaging procedure is most likely useful in evaluating gastrointestinal and urogenital diseases in GALS (6).

Kidney anatomy and urine formation in *L. fulica* were already described by Van Benthem Jutting in 1951 (7), Ghose in 1964 (8) and Martin et al. in 1965 (9). Van Benthem Jutting (7) showed the general anatomy on the basis of dissected *L.fulica* and described the kidney as one elongated organ, lying transversally in the left mantle cavity. Ghose (8) further described the kidney as single-lobed, soft and spongy, dull greyish and flat, adherent to the mantle and lying parallel to the heart. At one region the capsule of the kidney is fused with the pericardium and fluid from the pericardial chamber continually fills the kidney lumen through the reno-pericardial aperture.

The posterior part of the kidney receives blood from the reno-intestinal artery and the renal branches of the rectal artery, which form the afferent renal vessels. Blood then is returned to the rectal sinus, whereas blood from the anterior part of the kidney and its ventral surface are drained to the pulmonary vein through five or six vessels. The kidney cells absorb nitrogenous wastes from the blood which are discharged into the lumen of the kidney and then through the ureter, which opens in the pneumostome close to the anus. Arginase forms nitrogenous waste to urea which is further converted into uric acid (8). Further literature of the histology of the urinary system in Gastropods are provided by Dennis et al. (10).

The simple alimentary system of *L. fulica* consists of buccal mass with salivary glands, esophagus, two-chambered crop, stomach, and intestine. Crop, stomach, and intestine form an ‘U’, while stomach and first part of the intestine are nearly fully embedded in the large, spirally-twisted digestive gland. Finally, the intestine ends in the dorsal pneumostome (2, 11).

Here we report in detail the history of four adult *L. fulica* with apathy and esophagus protrusion or prolapse in three cases and edema and mantle collapse in another case. Renomegaly and/or kidney deposits could be demonstrated in radiographic and/or CT examinations in all four cases, while necropsy and histology revealing nephropathy could be performed in three cases.

## Case descriptions

Snail No. 1 and 4 were initially presented to the clinic as part of a diagnostic imaging study. They received radiographic examinations in laterolateral and dorsoventral view, as well as a multislice helical CT examination in 2015. The procedures were discussed and approved by the institutional ethics and animal welfare committee in accordance with GSP guidelines and national legislation (ETK-07109/2015). Snail No. 2 was presented as a patient at the clinic and snail No. 3 was referred via email.

### Snail No. 1

An adult Giant African Land Snail (*L. fulica*), weighing 437 g, was presented to the clinic as part of a diagnostic imaging study. The snail was kept together with another adult *L. fulica* in an aquarium (>75% humidity, 22.6–25°C) and fed daily with vegetables, animal protein (gammarus, dry cat food, tortoise stick food) and calcium.

The initial radiographs and a CT examination in 2015 revealed a mildly bumpy surface and mild thickening (max. 8 mm diameter) of the kidney as well as a focal, irregular hyperdense area (suspected deposit) at the distal end. The owner was advised to feed less animal protein, make warm baths and provide high humidity and fresh water *ad libitum*.

**Figure 1:**
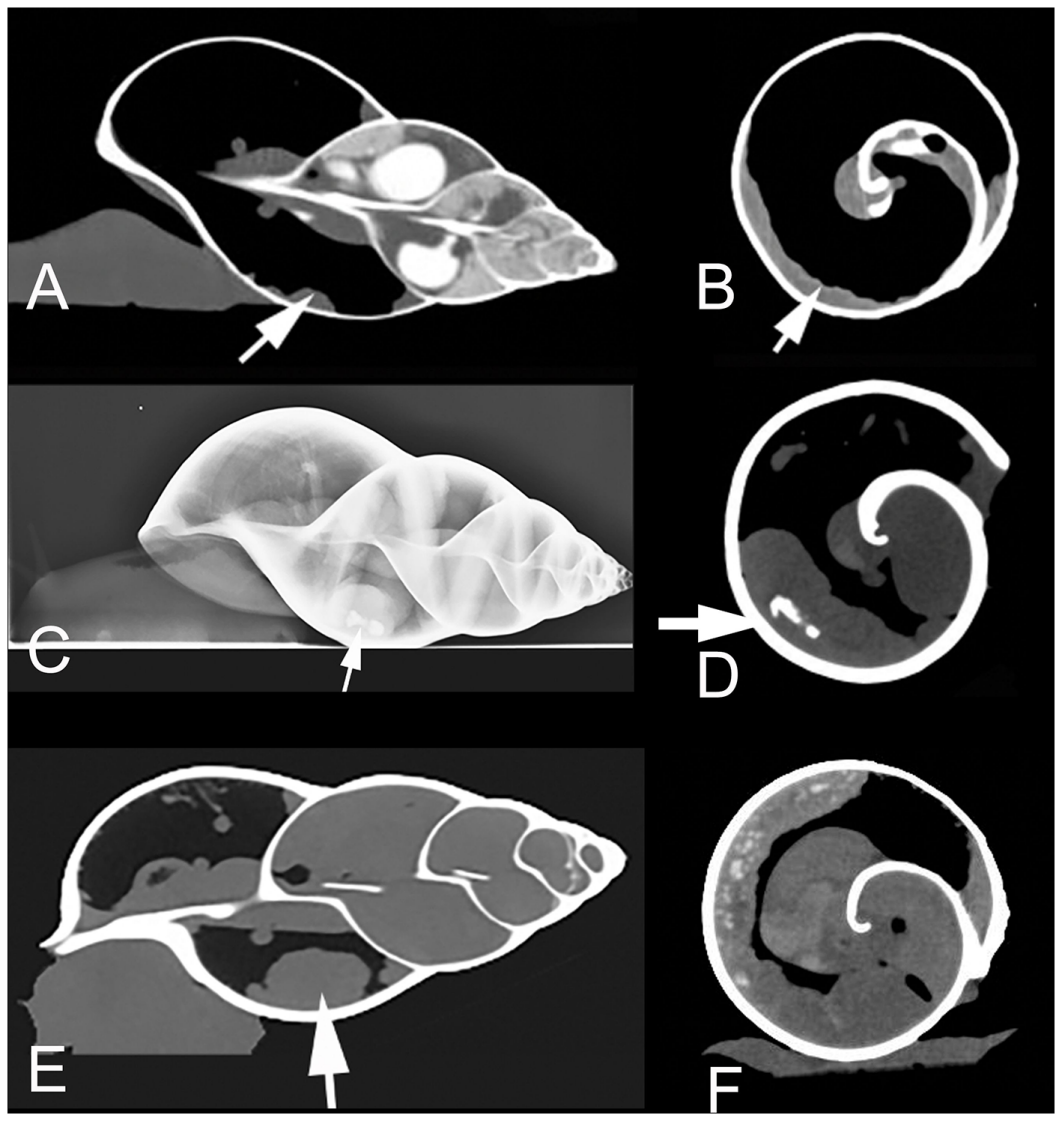
Sagittal **(A)** and transversal **(B)** CT (in soft tissue window) of a healthy Giant African Land Snail (GALS) for comparison, showing a slim, soft tissue dense, normal kidney (arrow), positioned along the wall of the first whirl of the shell. Lateral radiograph **(C)** as well as transversal **(D)** and sagittal (**E**, in modified bony window) CT of GALS No. 1 in October 2016. The kidney is indicated with arrows. The organ is severely enlarged with a bumpy surface. The kidney deposits (arrow in **(D)**) expanded even further till January 2017. Instead of measuring the absolute diameter of the kidney one may also note the relative size by observing the distance of the kidney surface to the central columella. The kidney here occupies more than 50% of the diameter of the whorl in **(D)**. **(F)** Transversal CT image (soft tissue window) of snail No. 2. The kidney presents from 6 to 12 o’clock along the inner contour of the shell. The kidney surface is bumpy and multiple hyperdense amorphous areas (kidney deposits) are visible.

In October 2016, the same snail, now weighing only 360 g, was presented to the clinic because of weakness and loss of appetite after development of a clutch. A follow up radiograph and CT-examination revealed that the kidney deposits had nearly doubled in size. The kidney surface showed severe irregularities and the diameter of the organ had massively increased (Figures 1C–E). The owner continued with conservative therapy as described above.

In January 2017 the snail was presented again because of an esophagus prolapse through the mouth, which regressed spontaneously. CT examination confirmed further worsening of kidney deposits and renomegaly. Two weeks later the owner reported a recurrent prolapse. Therefore the snail was euthanised in a local emergency veterinary clinic at night, but offered to the Small Animal Internal Medicine at Vetmeduni Vienna for further investigation.

**Figure 2:**
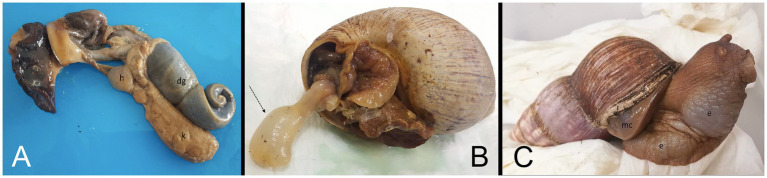
**(A)** Macroscopic picture of deceased Giant African Land Snail (GALS) No. 1 removed from her shell. Note the enlarged kidney (=*k*). *h* = heart, dg = digestive gland. **(B)** Macroscopic picture of deceased snail No. 2 with the prolapsed esophagus from the mouth, marked with an arrow. **(C)** Clinical picture of GALS No. 4 with mantle collapse (=mc) and severe edema (=e).

**Figure 3:**
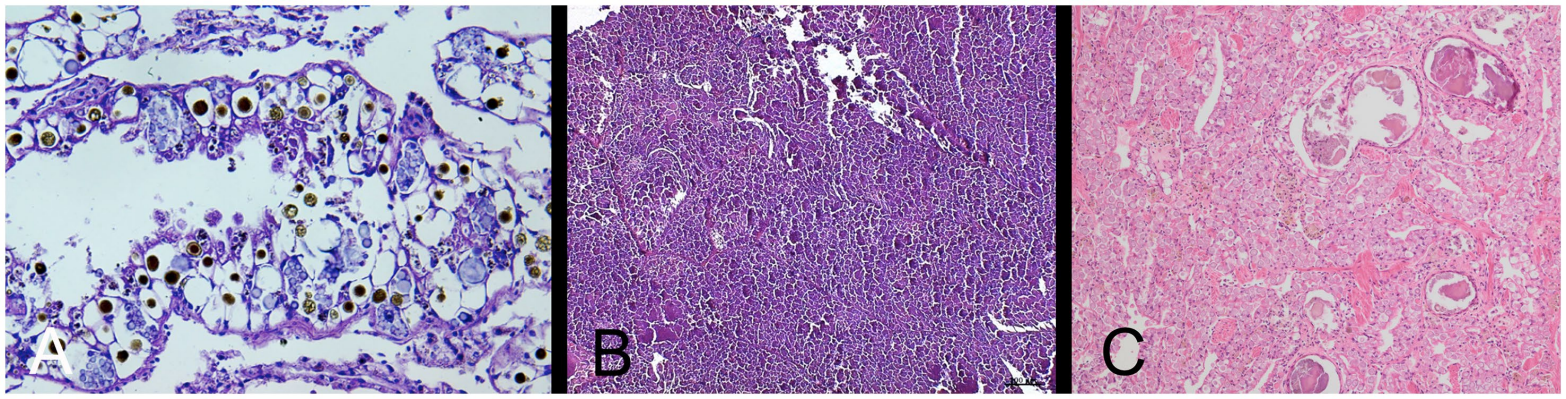
**(A)** Histopathological picture of the kidney of Giant African Land Snail (GALS) No. 1, showing nephropathy with edema and deposition of various spherocrystals. HE-stain, 40 x objective. (Exomed-Institute, Berlin, Germany). **(B)** Histopathological picture of the kidney of GALS No. 2, showing full degeneration of the kidney with no physiological nephrocytes but spherical to cloddy compartments. HE-stain, 10 x objective. (Exomed-Institute, Berlin, Germany). **(C)** Histopathological picture of the kidney of GALS No. 4, showing nephropathy with severe degeneration of the kidney and deposition of many spherocrystals as well as larger deposits, which seemed to consist of a mixture of accumulated excretions and cellular debris. HE-stain, 10 x objective (Vetmeduni, Vienna, Austria).

The deceased snail was removed from its shell for dissection. Figure 2A shows the internal organs of the snail (note the extremely enlarged kidney). Histopathology described metabolic kidney disorder with edema and deposition of spherocrystals (purines and purine bases) (Figure 3A).

### Snail No. 2

A second snail, weighing 283 g, was presented to the clinic because of weakness. The snail was kept together with other adult *L. fulicas* in an aquarium and fed with vegetables, bananas and animal protein (mealworms, tortoise stick food). The owner sprayed water twice a day into the terrarium, but no other water supply was provided. Temperature and air humidity were not measured.

A radiographic and a CT examination were performed. The kidney was severely enlarged with a bumpy surface and massive diffuse hyperdensities throughout the whole parenchyma (Figure 1F). The ureter appeared dilated. A few days later, the snail was presented again at the clinic because of prolapsed esophagus through the mouth (Figure 2B).

The snail was first anaesthetised with an overdose of ketamine and xylazine by intramuscular injection in the foot, followed by euthanasia with T 61^®^ (Intervet GmbH) when the animal was non responsive. Cessation of heartbeat was monitored by a Doppler ultrasound probe. The histopathological report described severe renomegaly, as well as a full degeneration of the kidney with deposition of various spherocrystals (purines, purine bases and urates) (Figure 3B).

### Snail No. 3

A third animal was referred via email to the clinic. The owner offered a detailed report about the husbandry and clinical signs of a 12 years old *L. fulica* as well as pictures of a beginning esophageal protrusion. The snail was kept together with two other adult *L. fulicas* in an aquarium. All snails were adopted by the owner when they were already 6 years old from an experimental station with irregular food and water supply. They were now fed with vegetables, fruits, like bananas, and animal protein (gammarus). The owner sprayed water into the aquarium, but had problems to keep the humidity at an appropriate level. Temperature was about 23°C in the aquarium.

Performance of radiographs was advised in a phone call. A local veterinarian provided laterolateral, dorsoventral, and craniocaudal views. Severe renal enlargement was diagnosed with moderately bumpy surface of the kidney (similar to Figure 1C). The owner continued with conservative treatment. The snail lived for two more years and was not available for pathological evaluation.

### Snail No. 4

This *L. fulica* was held by the same owner as Snail No. 1. The initial diagnostic imaging examinations were unremarkable.

In April 2022, the snail was presented to the clinic because of a mantle collapse and edema in the regions of the foot and head (Figure 2C). The snail was weighing 453 g. CT examination revealed renomegaly with a heterogeneous parenchyma and several elongated hyperdense areas within the parenchyma (similar to Figures 1D–E). Some other organs were hypodense and most of them not even differentiable due to the severe edema.

The snail was euthanised in accordance with the owner as described in case 2 and then send to pathology. Histopathology of the kidney revealed nephropathy with severe degeneration of the kidney and deposition of many spherocrystals as well as larger deposits, which seemed to consist of a mixture of accumulated excretions and cellular debris. The connective and muscular tissue of the foot was interspersed with a multitude of slit-like empty spaces, interpreted to be extracellular edema (Figure 3C).

## Discussion

Kidney disorders are common in exotic animals, but clinical signs are often non-specific and not recognized until an advanced state of illness. Giant African Land Snails are frequently held as pets in Europe and when their snails are ill, owners seek for veterinarian help. In our cases, all snails showed apathy and three of them (No. 1–3) had esophagus prolapse or protrusion. One snail (No. 4) suffered from severe edema and mantle collapse.

Based on our previous study results about diagnostic imaging in GALS (6), we chose radiographic and CT examinations for further evaluation. We could visualize the snail kidneys with these imaging techniques as one elongated, smoothly bordered organ of soft tissue density, lying transversally in the left mantle cavity along the shell in healthy animals (Figures 1A,B).

Renomegaly and/or kidney deposits could be demonstrated by diagnostic imaging in all four cases shown here. Furthermore follow-up imaging showed progressive worsening of kidney disease in snail No.1 and 4.

An interesting finding is, that esophagus prolapse or protrusion appeared in three out of four snails (No. 1–3). Possibly the severely enlarged kidney exerts pressure on the upper gastrointestinal tract leading to esophagus prolapse through the mouth. Snail No. 4 showed massive edema which led to a mantle collapse and the snail detached from her shell. Organ prolapse through the mouth as well as mantle collapse are feared conditions among snail owners with poor prognosis because these conditions are often caused by other underlying diseases.

Finally, three snails (No. 1, 2, and 4) were euthanised because of their severe clinical symptoms and sent to necropsy and/or histopathology which confirmed nephropathy in different stages with renomegaly, renal edema, kidney degeneration and various kidney deposits. The deposited spherocrystals most likely represent accumulated purines, purine bases and urates. Similar histological pictures with remarkably fewer and reversible depositions are visible in healthy kidneys of land snails during resting periods (personal communication with Dr. Mutschmann, Exomed). In our cases (No. 1–2, and 4) massive depositions in active periods most likely indicate severe metabolic disorders. Snail No. 2 additionally showed full degeneration of the kidney with no physiological nephrocytes left but spherical to amorphous, fragmented material indicating end-stage renal disease.

Kidney deposits in CT showed urate density of 150 to bone density of 1,500 Hounsfield Units (HU). We compared the density of the deposits in the snail kidneys with urates of tortoise urine (150–200 HU) to confirm the former. Unfortunately kidney deposits with mineral density in No. 1 and 4 could not be histologically confirmed as calcifications. Perhaps the affected part was not trimmed and thus missed during histopathological examination. Similar mineral dense renal deposits can be found in CT scans of tortoises suffering from gout or other severe kidney disease and in these cases the kidney deposits could be histologically confirmed as kidney calcifications (personal observations).

Urinary diseases are often the result of inadequate husbandry and diet and it is reported that dehydration and excess dietary protein could increase the risk of renal disorders in reptiles (12, 13). A previous study about *L. fulica* showed a wide range of food spectrum for this terrestrial snail including plants, but also dead decomposed animal tissue. However, it is also described that these snails can survive without food for a very long time (2). Srivastava (2) also reported that *L. fulica* is more active under humid conditions, while it hibernates during inappropriate conditions like dry periods. In our case reports, large amount of protein intake, the overfeeding and the inadequate water supply may have led to nephropathy.

Therapy of kidney disease is often only supportive in exotic species and optimization of husbandry like proper hydration is one of the most important aspects (13). Therefore the owners of the snails in these cases were advised to provide warm water baths, high humidity and fresh water *ad libitum* to the snails. Unfortunately kidney disease is usually a chronic, progressive process.

Although invertebrates are becoming increasingly popular as pets and some comprehensive books and papers on veterinary medicine in invertebrates exist, still little is known about land snail diseases and their therapy. Studies on healthy kidneys of land snails during active and resting periods as well as studies on kidney diseases are missing. Therefore we hope to stimulate veterinary research in these species with our first case reports in *L. fulica*s with kidney diseases.

## Data availability statement

The original contributions presented in the study are included in the article/supplementary material, further inquiries can be directed to the corresponding author.

## Ethics statement

The procedures were discussed and approved by the institutional ethics and animal welfare committee in accordance with GSP guidelines and national legislation (ETK-07109/2015). Written informed consent was obtained from the owners for the participation of their animals in this study.

## Author contributions

SS-U and MG contributed to conception and design of the study. SS-U collected the cases and wrote the first draft of the manuscript. SS-U, MG, and BR wrote sections of the manuscript. FM and BR were responsible for pathological and histological analysis and interpretation of results in two (FM) and one (BR) case. MG made the imaging part in all cases and SS-U the clinical part in all cases. All authors contributed to the article and approved the submitted version.

## Conflict of interest

The authors declare that the research was conducted in the absence of any commercial or financial relationships that could be construed as a potential conflict of interest.

## Publisher’s note

All claims expressed in this article are solely those of the authors and do not necessarily represent those of their affiliated organizations, or those of the publisher, the editors and the reviewers. Any product that may be evaluated in this article, or claim that may be made by its manufacturer, is not guaranteed or endorsed by the publisher.

## Footnotes

^1^www.molluscabase.org
